# Critical role of molecular test in early diagnosis of gastric tuberculosis: a rare case report and review of literature

**DOI:** 10.1186/s12879-019-4225-7

**Published:** 2019-07-05

**Authors:** Jun Ma, Hongyun Yin, Huikang Xie

**Affiliations:** 1 Clinic and Research Center of Tuberculosis, Shanghai Key Lab of Tuberculosis, Shanghai Pulmonary Hospital, Tongji University School of Medicine, Shanghai, 200433 China; 2 Department of Tuberculosis and Shanghai Key Lab of Tuberculosis, Shanghai Pulmonary Hospital Affiliated to Tongji University, 507 Zhengmin Road, Shanghai, 200433 China; 3 Pathology department, Shanghai Pulmonary Hospital Affiliated to Tongji University, 507 Zhengmin Road, Shanghai, 200433 China

**Keywords:** Gastric tuberculosis, Diagnosis, Polymerase chain reaction

## Abstract

**Backgroud:**

Early diagnosis of gastric tuberculosis is often challenging because the disease is very rare and its clinical manifestation is nonspecific and misleading. To raise the awareness and emphasize early diagnosis of gastric tuberculosis, we present a case of gastric tuberculosis secondary to pleural and pulmonary tuberculosis.

**Case presentation:**

A 26-year-old woman complained gastric pain for 1 month but showed no other symptoms, who had no previous exposure to tuberculosis.Gastric stromal tumor was originally suspected. However, the pathology of her gastroscopic biopsy of the gastric lesion showed granulomatous lesions and caseating necrosis. Gene sequencing of the biopsy specimen identified deoxyribonucleic acid fragment of *Mycobacterium tuberculosis*. Chest computed tomography scan revealed nodular shadows in the lesser curvature soft tissue of the stomach, patchy densities and calcified nodular shadows in the upper right lung, bilateral pleural thickening, and calcified pleural nodules. Thus, the diagnosis was gastric tuberculosis secondary to pulmonary and pleural tuberculosis. The patient was hospitalized and treated with the antituberculosis therapy for 1 week. After discharged from the hospital, the patient continued routine antituberculosis therapy for 18 months and was follow-up was normal.Literature search found 22 cases of gastric tuberculosis reported from 2000 to 2016. Review of the 22 cases suggested that polymerase chain reaction has been increasingly used in the recent years in addition to the conventional histopathological and bacteriological approaches.

**Conclusion:**

Clinical presentation of gastric tuberculosis is not specific.When granuloma or caseation is detected on biopsy in patients who are suspected of having gastric malignancy or acid peptic diseases, polymerase chain reaction for *Mycobacterium tuberculosis* could be used as an available and sensitive diagnostic test in addition to pathology, acid-fast bacilli smear staining and culture.

## Background

The gastrointestinal tract is the sixth most common location for extrapulmonary tuberculosis (TB) [1]. However, gastric TB is very rare because the unique characteristics of the stomach, such as gastric acid, fast gastric emptying, and scarcity of lymphatic tissue in the gastric wall, may protect the organ from TB [2]. Subei et al. have reported that the incidence of gastric TB is 0.03–0.21% in all routine autopsies [3]. In China, the incidence appears higher. Yang et al. found that the incidence of gastric TB in Chinese patients undergoing gastrectomy between 1962 and 2002 in 10 hospitals in northeastern China was 0.37% [4].

Gastric TB often develops secondary to pulmonary TB [5]. Previous reports suggest that:the frequency of gastric tuberculosis is related to the severity of pulmonary involvement. In the study of Mitchell and Bristol, it was present in 1% of patients with minimal pulmonary tuberculosis, 4.5% in those with moderately advanced pulmonary tuberculosis, and 25% in those with advanced disease [6].Nevertheless, primary gastric TB has been sporadically reported during the past 16 years [7–13]. Here, to promote the awareness of gastric TB and emphasize accurate diagnosis and prompt treatment for the disease, we present a case of gastric TB secondary to pleural and pulmonary TB and review previous cases that were reported between 2000 and 2016.

## Case presentation

**Fig. 1:**
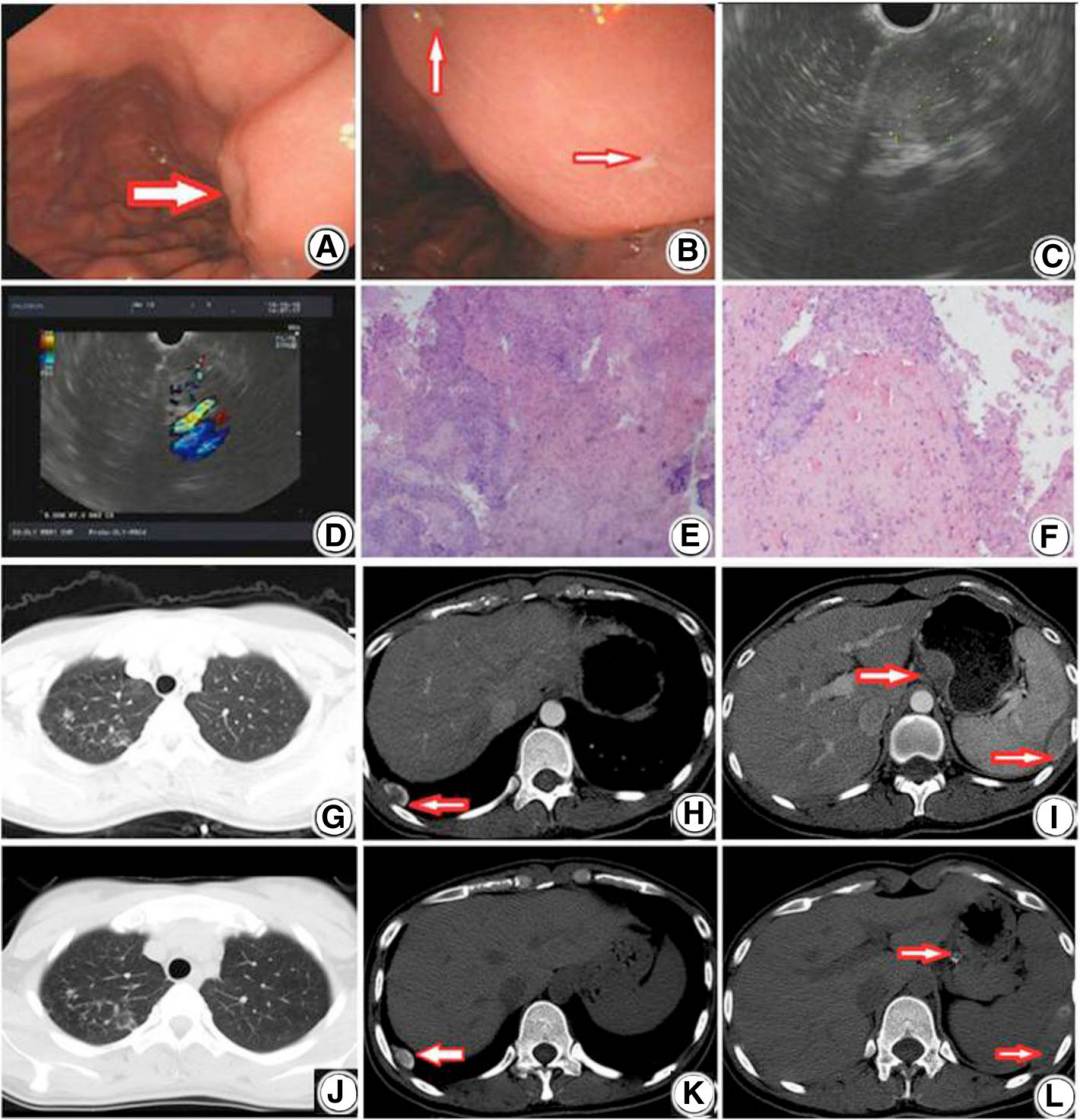
Images of gastroscopy, endoscopic ultrasound, Haematoxylin Eosin staining, Chest CT scan. **a** and **b**. Images of gastroscopy showing a hemispherical bulge with smooth surface (**a**) and two small ulcers on the posterior wall of the lesser curvature of the stomach (**b**). **c** and **d**. Images of endoscopic ultrasound showing hypoechoic masses with a size of 26 mm × 21.5 mm (**c**) and ununiform echo with some area of strong echo inside the lesion, and rich blood flow inside the lesion (**d**). **e** and **f**. Images of Haematoxylin & Eosin staining showing patches of caseating necrosis and granulomatous inflammation (**e**: 40x, **f**: 100x). **g**-**i**. Image of Chest CT scan at hospital admission (December 2013) showing high densities and patchy nodules in the upper right lung (**g**), calcified pleural nodules in the lower right pleural (**h**), and soft tissue nodules in the lesser curvature of the stomach (**i**). **j**-**l**. Image of chest CT at the follow-up (May 2015) showing that the patchy nodules in the upper right lung were partially absorbed (**j**), smaller pleural nodules (**k**), and substantially smaller nodules with some calcification in the lesser curvature of the stomach and calcified pleural nodules in the lower left pleural (**l**).

A 26-year-old woman patient was admitted to Shanghai Pulmonary Hospital on December 13, 2013 for constant gastric pain for 1 month. One month before the hospital admission, the patient started to experience gastric pain accompanied with acid reflux for no apparent cause. The pain became worse on an empty stomach. The patient did not present swallowing difficulties, belching, nausea, vomiting blood, black stool, fever, fatigue, diarrhea, or tenesmus at hospital admission. The patient had no previous exposure to tuberculosis.Gastroscopy revealed a hemispherical bulge with smooth surface (Fig. 1a) and two small ulcers on the posterior wall of the lesser curvature of the stomach (Fig. 1b). Endoscopic ultrasound showed hypoechoic masses around the lesion (Fig. 1c), ununiform echo with some area of strong echo inside the lesion, and rich blood flow inside the lesion (Fig. 1d). The lesion was located in the fourth echo layer and showed a dimension of 26 mm × 21.5 mm (Fig. 1c). The patient was originally suspected to have gastric stromal tumor. The pathology of gastroscopic biopsy displayed acid-fast staining negative, reticular fiber staining negative, periodic acid Schiff staining negative, and ammoniacal silver staining negative. Haematoxylin & eosin staining of the gastroscopic biopsy specimen showed patches of caseating necrosis and granulomatous inflammation (Fig. 1e and f). Gene sequencing by polymerase chain reaction(PCR)analysis of the gastroscopic biopsy specimen found *Mycobacterium tuberculosis* (M. TB) deoxyribonucleic acid (DNA) fragments.

Chest computed tomography (CT) scan demonstrated: 1) patchy densities and calcified nodular shadows in the upper right lung (Fig. 1g), suggesting a possible previous lung TB; 2) calcified pleural nodules in the lower right pleural (Fig. 1h); 3) nodular shadows in the lesser curvature soft tissue of the stomach (Fig. 1i). Chest CT-guided percutaneous needle biopsy on the pleural nodules found necrotic tissue and inflammatory cells, The acid-fast stain smear was negative, Along with other findings in chest CT and gastric pathology and positive TB-PCR, these pathologic changes may be in favor of TB pleural involvement. Electronic Bronchoscopy showed normal lumen and mucosal membrane. The patient did not have a previous diagnosis of TB. She had normal routine blood and chemistry test results. Tuberculin skin test (TST) showed an induration of 22 mm × 18 mm. M. TB antibody test and T-SPOT.TB test (A30 and B10) were positive. Based on these clinical data, the patient was diagnosed as gastric TB. First acid-fast bacilli smear and culture tests were negative.

The patient was hospitalized and underwent the systematic antitubercular therapy(ATT) HREZ for 1 week (0.3 g once daily oral isoniazid., rifampicin 0.45 g once a day on an empty stomach, 0.75 g once daily oral ethambutol, pyrazinamide orally, 0.5 g three times a day. Oral protection of liver is also given). The patient insisted on an outpatient review every month, with routine blood tests and liver and kidney function tests. After 3 months of anti-tuberculosis treatment, the patient developed gastrointestinal reaction and blurred vision, and the adjustment programme was HRptZLfx,(Isoniazid 0.3 g 1 day orally, rifapentini 0.45 a week twice fasting, pyrazinamide 0.5 g a day three times, levofloxacin 0.5 g 1 day orally). The above side effects were alleviated.After 6 months of treatment, levofloxacin was discontinued and continued HRptZ treatment. She had normal monthly reexamination of the normal liver and kidney function, and acid-fast bacilli smear and culture tests were negative for every 3 months. The erythrocyte sedimentation rate and C reactive protein were negative before the drug withdrawal. Her gastroscopy showed normal results. Her chest CT scan revealed calcified nodules in the upper right lung (Fig. 1j), bilateral pleural thickening (Fig. 1k), calcified pleural nodules (Fig. 1l), and substantially smaller nodules with some calcification in the lesser curvature (Fig. 1l) compared with the chest CT scan results on the hospital admission (Fig. 1g-i). Her sputum acid-fast bacilli smear and culture tests in March and May 2015 showed negative results. The antitubercular therapy was ended in May 2015. Her total treatment course was 18 months.

### PCR approach

Deparaffinisation and DNA extractionTissue sections 5 μm thick were cut from paraffin blocks, and 10 sections were placed in 1.5 ml sterile EP tube. The tissues were deparaffinised by adding 1 ml of xylene to each microtube and the samples were mixed gently for 20 min at 65 °C. The samples were then centrifuged at 13000 rpm for 2 min and the supernatants were discarded. Residual xylene was removed by washing the samples twice with 1 ml of 100% ethanol for 5 min and centrifuging at 13000 rpm for 2 min. The pellets were dried in a speed vacuum, and 200 μl of FTL and 25 μl of OB Protease (OMEGA). Genomic DNA was isolated using the E.Z.N.A.TM FFPE DNA kit from Omega Biotek Inc. (Norcross, GA, USA) according to the protocol provided by the manufacturer. The contents and purity of the extracted DNA were assayed by measuring absorbance at 260 and 280 nm using a spectrophotometer.PCR amplificationIS6110 gene is specific for MTBC and 16SrRNA gene is specific for mycobacterium genus. The following primer sets were used for PCR: IS6110 (sense 5 ‘-CCTGCGAGCGTAGGCGTCGG-3′, anti-sense 5′- CCTGCGAGCGTAGGCGTCGG-3′); the product size of IS6110 was 123 bp a. Reactions were run in 25ul volumes containing 2ul of sample DNA. The remaining volumes was added using PCR master Mix (2×) from Thermo according to the protocol provided by the manufacturer. The amplification program was consisted of initial denaturation at 95 °C for 5 min, followed by 35 cycles of denaturation at 94 °C for 45 s, annealing at 60 °C for 30s, extension at 72 °C for 45 s, and a final extension at 72 °C for10 min. The PCR products were analyzed by 2% agarose gel electrophoresis [14].

### Summary of previously reported cases from 2000 to 2016

We searched PubMed for the keyword “gastric tuberculosis” and found that 22 cases of gastric TB were reported from 2000 to 2016 [7–13, 15–25]. The summary of these cases is displayed in Table 1. The patients were 21 to 80 years of age, and the mean age was 38.6 ± 15.3 years. The average history of disease were 6.4 ± 6.06 months. The shortest is 1 month. Of the 22 cases, 45.5% (10/22) were men; 59.1% (13/22) were primary gastric TB; 31.8% (7/22) had gastric outlet obstruction (Table 1). Two cases were gastric TB with concomitant cancer [18, 24]. PCR was commonly used to diagnose gastric TB since 2011 (Table 1) [8, 9, 15–18]. Most cases presented caseating granuloma in endoscopic biopsy specimens or surgical specimens (Table 1). Notably, a few cases did not show confirmed diagnosis of gastric TB but responded well to empiric ATT (Table 1) [9, 19]. All the patients survived and respond well to routine ATT.

**Table 1:** Summary of previously reported cases from 2000 to 2016

Authors	Year	Age	Sex	Clinical feature	Diagnostic approach	Treatment duration	strategy (combination of anti-TB drugs)
Arabi [7]	2015	54	M	Primary gastric TB with outlet obstruction	Histopathological confirmation	NA	NA
Yaita Hiroki [15]	2014	60	M	Gastric TB with systemic ymphadenopathy	PCR of biopsy specimen was positive for M. TB	NA	NA
Moghadam [8]	2013	43	M	Primary gastric TB mimicking gastric cancer	PCR of surgical specimens was positive for M. TB.	6 months	2HREZ/4HR
Ecka [16]	2013	31	M	Isolated gastric TB with outlet obstruction	PCR of tissue biopsy was positive for M. TB.	9 months	2HREZ/7HR
Lim [15]	2013	38	F	Gastric TB with a huge abdominal mass	Endoscopic biopsy specimen was positive on acid-fast bacillus staining. PCR of the biopsy specimen was positive for M. TB.	12 months	AmMfxPtoCsZ
Kang [18]	2012	54	F	Gastric cancer concomitant gastric TB	Tuberculosis PCR of the gastric mucosa and omental lymph nodes was positive for M. TB.	NA	NA
Ishii [9]	2011	39	F	Primary gastric TB presenting as non-healing ulcer and mimicking Crohn’s disease	Respond to empiric ATT. All the test including PCR showed negative for M. TB.	10 months	2HREZ/8HRE
Mukhopadhyay [10]	2010	30	F	Isolated gastric TB	Histopathology revealed granulomatus inflammation of M. TB	6 months	2HREZ/4HR
Bandyopadhyay [19]	2010	na	na	Gastric TB with outlet obstruction	Respond to empiric ATT	NA	NA
Baylan [20]	2009	80	F	Primary gastric TB	PCR of biopsy specimen was positive for M. TB	6 months	2HREZ/4HR
Khan [11]	2008	29	M	Primary gastric fundus TB	Endoscopic biopsy showed caseating granulomas with acid-fast bacilli in the ulcerative mass.	6 months	6HREZ
Talukdar [21]	2006	30	F	Gastric TB presenting as linitis plastica and outlet obstruction	Endoscopic biopsy specimens showed caseating granulomas and positive for acid fast bacilli staining.	NA	HREZ
Kim [22]	2005	21	F	Gastric TB presenting as a submucosal tumor	Histopathologic examination of the surgical specimens revealed chronic granulomatous inflammation with caseation necrosis. PCR for M. TB with the surgical specimens was positive.	3 months	3HRE
Sharma [23]	2004	21	F	Gastric TB with a perforation	Histopathological examination revealed tuberculous granulation and acid-fast bacilli in the ulcer.	17 months	NA
Amarapurkar [12]	2003	32	F	Primary gastric TB with outlet obstruction	Lymph node biopsies showed positive for acid-fast bacilli staining.	9 months	2HREZ/7HR
		53	M	Primary gastric TB with outlet obstruction	Histopathology of the lymph node revealed caseating granuloma	9 months	2HREZ/7HR
		23	F	Primary gastric TB with outlet obstruction	Histology demonstrated caseating granuloma with the presence of acid fast bacilli.	9 months	2HREZ/7HR
		32	M	Primary gastric TB	Endoscopic biopsy revealed caseating epitheloid granuloma with Langhan’s giant cells.	9 months	2HREZ/7HR
		30	M	Primary gastric TB	Endoscopic biopsy showed multiple tubercular caseating granulomas.	9 months	2HREZ/7HR
Khan [24]	2003	na	na	Gastric TB with concomitant stromal tumor of stomach		NA	NA
Wig [13]	2000	25	M	Isolated gastric TB presenting as massive hematemesis	Histopathological examination	NA	NA
Chetri [25]	2000	46	M	Gastric TB presenting as non-healing ulcer	Endoscopic biopsy specimens showed caseating granulomas and positive for acid fast bacilli staining.	NA	NA

*ATT* antitubercular therapy, *PCR* polymerase chain reaction, *M. TB Mycobacterium tuberculosis*, *H* isoniazid, *R* rifampicin, *E* ethambutol, *Z* pyrazinamide, *Am* kanamycin, *Mfx* moxifloxacin, *Pto* prothionamide, *Cs* cycloserin, *NA* not avalaible

## Discussion and conclusion

Gastric TB likely occurs at the pyloric antrum because of an increased amount of lymphoid tissue in the pyloric antrum compared with other area in the stomach. Although normal physiological features of the stomach may have some protective effects, abnormal gastric conditions, such as injury, erosion, and/or ulcer in the gastric mucosa, a reduction in gastric acid, and delayed gastric emptying may extend the residence time of M. TB in the stomach and thus increase the risk for gastric TB. Four possible routes of infection have been proposed: 1) a direct gastric mucosal infection, for example, swallowing M. TB-contaminated sputum or food; 2) hematogenous dissemination of M. TB; 3) M. TB-spread via lymphatic system; 4) infection from adjacent M. TB-infected abdominal organs, such as pancreatic and/or splenic TB [2]. Gastric TB is often thought to develop secondary to pulmonary or other extrapulmonary TB [5]. In the current study, our patient’s chest CT scan showed high-density patchy calcified nodules in upper right lung and calcified pleural nodules, suggesting that her gastric TB appeared to be secondary to pulmonary and pleural TB for years or more. Thus, screening for possible pulmonary and extrapulmonary TB could facilitate an accurate diagnosis of gastric TB.

The current study also found that 59.1% of the previous cases reported between 2000 and 2016 were primary gastric TB. Diagnose of primary gastric TB is often challenging because the clinical presentation of gastric TB varies greatly and lacks specific features. Salpeter et al. suggested that only approximately 50% of primary or isolate gastric TB might be diagnosed accurately [26]. The most frequent complaint associated with gastric TB is chronic epigastric pain, which usually lasts couple months. Patients often also present other symptoms, such as vomiting, pyloric obstruction, diarrhea, constipation, fever, weight loss, and fatigue. In addition, patients with gastric TB frequently develop gastric outlet obstruction. In the current study, we found 31.8% (7/22) of previous cases had outlet obstruction [2, 7, 12, 16, 19, 21]. Notably, in the current study, gastric pain was the only obvious complaint of our patient.

Gastric TB is sometimes misdiagnosed as gastric cancer or tumor, because its clinical presentations resemble those of gastric cancer, particularly because of the presence of an epigastric mass in both conditions. Kim et al. have demonstrated a case of gastric TB presenting as a submucosal tumor, which was originally suspected as stromal tumor but later confirmed as gastric TB based on the chronic granulomatous inflammation with caseation necrosis in the resected tissue and positive PCR for M. TB of the resected tissue [22]. In the current study, gastric stromal tumor was originally suspected. Concomitant gastric cancer and TB may even further complicate the diagnosis of gastric TB. Kang et al. have reported a case of coexisting TB and gastric adenocarcinoma [18], and Khan et al. have shown a simultaneous stromal tumor of stomach and gastric TB [24]. Kim et al. have recommended that gastric TB should be suspected if patients exhibit TB in other organs, positive tuberculin test, an obvious abdominal mass, fistula formation, and duodenum involvement [22].

Early diagnosis of gastric TB is critical to improve outcomes. If left untreated, gastric TB could deteriorate and ultimately lead to gastric perforation. Sharma et al. have reported a patient with tuberculous gastric perforation, who received emergency distal gastrectomy and postoperative ATT and survived eventually [23]. However, the four cases of tuberculous gastric perforation, which were reported between 1948 and 2003, all died [24]. Currently, histological, bacteriological, and molecular biological approaches have been used to diagnose gastric TB.

Gastroscopic observation and biopsy remain the most commonly used approaches to diagnose gastric TB. The presence of caseating granuloma or positive acid-fast bacilli staining on the lesion tissues are usually considered as valid clinical evidence for gastric TB [5]. However, acid-fast bacilli staining sometimes show false negative results. In fact, our patient showed negative acid-fast bacilli staining. Ishii et al. have demonstrated a patient with primary gastric TB showing negative acid-fast bacilli staining, but the patient responded to empiric ATT effectively [9]. Baylan et al. have also shown that a patients presented negative acid-fast bacilli staining and negative bacterial culture of the biopsy specimen, whereas had positive PCR for M. TB [20]. Moreover, collecting sufficient and accurate specimens during gastroscopic biopsy sometime is challenging because granulomas are often located underneath the gastric mucosa, which is difficult to reach during a gastroscopic biopsy procedure. Thus, most patients may have to undergo surgical intervention when gastroscopic biopsy fails to support an accurate diagnosis. The subsequent histopathological examination of the surgical samples can usually confirm a diagnosis of gastric TB.

Notably, molecular biological technique, such as polymerase chain reaction (PCR) and DNA sequencing, have been increasingly used in the recent years [8, 9, 15–18]. In 2005, the report by Kim et al. appeared to be the first case of using PCR to detect M.TB in surgical specimens [22]. In 2009, Baylan et al. first reported a successful application of PCR on gastroscopic biopsy specimen to diagnose gastric TB [20]. PCR was used to diagnose gastric TB in 7 of the 10 cases that were reported from 2009 to 2016 [7–10, 15–20]. In the current study, gene sequencing of gastroscopic biopsy specimen showed positive for M. TB. PCR appears to be more frequently used to diagnose intestinal TB [27–29]. Balamurugan et al. found that the sensitivity and specificity of fecal PCR to diagnose intestinal TB were 88.8 and 100%, respectively [28]. The diagnostic performance of PCR in gastric TB remains to be determined. In the current study, we found that PCR of biopsy or surgical specimens was performed in 8 cases; 7 of them (87.5%) showed positive M.TB; only one case had false negative result [9]. False negative is often caused by insufficient tissue samples. Therefore, it is very important to obtain high-quality specimens. It is often relatively easy to get pathological diagnosis and PCR amplification. However, the diagnosis rate of gastric tuberculosis is lower in gastroscope. The reason may be that the granuloma is often confined to the mucous membrane and the gastroscopy biopsy is mostly taken from the surface of the lesion, and it is not easy to reach the central position of the lesion. Fewer effective specimens were obtained, so false negative results were often obtained.In this case, endoscopic ultrasonography was performed at the same time in the endoscopic examination, and the biopsy site was accurately positioned to obtain high quality pathological specimens. Compared with other diagnostic methods, PCR appears to provide results rapidly, and thus may be a promising method for early diagnosis of gastric TB.

Clinical presentation of gastric tuberculosis is not specific.When granuloma or caseation is detected on biopsy in patients who are suspected of having gastric malignancy or acid peptic diseases, PCR for *Mycobacterium tuberculosis* could be used as an available and sensitive diagnostic test in addition to pathology, acid-fast bacilli smear staining and culture.

## Endnotes

Yes.

## Data Availability

The datasets used and/or analysed during the current study are available from the corresponding author on reasonable request.

## Abbreviations

ATT: Antitubercular therapy
CT: Computed tomography
DNA: Deoxyribonucleic acidcomputed tomography
E: Ethambutol
H: Isoniazid
Lfx: Levofloxacin
M. TB: *Mycobacterium tuberculosis*
PCR: Polymerase chain reaction
R: Rifampicin
Rpt: Rifapentini
TB: Tuberculosis
TST: Tuberculin skin test
Z: Pyrazinamide
